# 

**Published:** 1908-07-11

**Authors:** 


					ice
>h TiHfe MODERN Telephone Number :
2734 Gerrard.
legraphlc Address i i _ -? \] A T ,
0?pedale, Londoji. || medical journ
TT
Mfffi
W SERIES. No. 70, Vol. III. SatttRDAY
^No-1142. Vol. XLIV. oATUKDAi,
July 11th, 1908.
PRICE THREEPENCE).
^ '1'elc.a.cU J

				

